# Identification of Sensory Drivers of Liking of Plant‐Based Milk Using a Novel Palm Kernel Milk—The Effect of Reformulation and Flavors Addition Through CATA and PCA Analysis

**DOI:** 10.1002/fsn3.4719

**Published:** 2025-01-12

**Authors:** Shi‐Cheng Tong, Lee Fong Siow, Yee‐Ying Lee

**Affiliations:** ^1^ School of Science Monash University Malaysia Bandar Sunway Selangor Malaysia; ^2^ Monash Industry Plant Oils Research Laboratory (MIPO) Monash University Malaysia Bandar Sunway Selangor Malaysia

**Keywords:** CATA analysis, flavor, palm kernel, plant‐based milk, sensory analysis

## Abstract

There is a growing demand for a plant‐based diet (meat analogue/plant‐based milk) due to an increase in awareness towards health issues, environmental sustainability, and animal ethical issues. The replacement of dairy has recently been one of the market efforts to fulfill such demand. Yet, challenges arise when consumers are reluctant to accept plant‐based milk (PBM) due to the mismatch of organoleptic profile between PBM and the actual dairy. Hence, this study aims to understand the sensory drivers of liking in PBM through the development of a novel palm kernel milk (PKM 1.0). Furthermore, the study also aimed to examine the effect of reformulation (PKM 2.0) and flavor addition on sensorial acceptability improvement. Results showed that PKM 2.0 appeared similar to almond milk but is nutritionally denser like soymilk and oat milk. An acceptance score of 5.17 out of 9 was obtained for PKM 2.0 which is only slightly lower than 6.18 out of 9 and 6.36 out of 9 for oat milk and soymilk, respectively. Introducing flavors significantly improves the sample acceptance and reduces “bland” attributes. A high acceptance score of 7.24 out of 9 was obtained for chocolate‐flavored PKM along with a strong correlation with “rich”, “sweet”, and “creamy” attributes. A correlation matrix showed that “smooth”, “sweet”, “roasted”, “creamy”, and “rich” are the attributes the consumers desired.

## Graphical abstract

Correlation between hedonic analysis and CATA analysis helps to identify preferred sensory traits among different plant‐based milks. The sensory traits preferred by consumers are “roasted”, “sweet”, “smooth”, “rich”, and “creamy.” Addition of flavors significantly enhanced the acceptability of plant‐based milk.

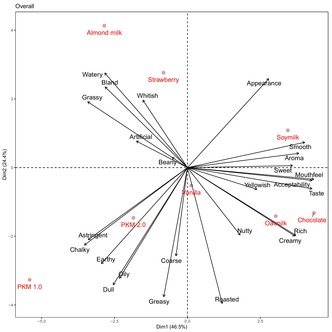

## 1 Introduction

Demand from consumers for a “dairy‐free” dairy beverage or a plant‐based dairy alternative was getting recognized due to rising awareness towards personal health, animal cruelty and environmental sustainability (Haas et al. 2019). Nevertheless, consumers, too, are rejecting most plant‐based milk (PBM) on the market due to a bad experience with consuming PBM or a negative impression towards the taste of PBM (Collier et al. 2023). Undoubtedly, there is a huge difference between the taste of dairy milk and PBM. Hence, the challenge lies in improving the sensory profile of PBM for higher consumer acceptance while maintaining the potential health benefits of PBM.

Although marketed as a replacer for dairy milk, the most common sensory traits associated with PBM are “watery”, “grassy”, “beany”, and “astringent” (Vaikma et al. 2021; Yao et al. 2022). Among those traits, “astringent” is generally considered a negative trait in most products that drive consumers away (Lesme et al. 2024) while consumers often prefer a creamy mouthfeel for dairy (Palacios et al. 2009). The characteristic “beany” and “grassy” flavor of PBM, on the other hand, can have either positive or negative effects depending on the consumers' cultural background (Yang et al. 2016). Nevertheless, not all PBM portray similar sensory profiles. The organoleptic properties of a PBM are highly dependent on the raw materials, functional ingredients, and processing technology used (Jemma et al. 2023). The diversity of PBM remains an attractive feature for consumers who are willing to explore and try new products in their daily diet (Basu 2022). Thus, the potential future direction of improving the acceptability of PBM likely lies within eliminating the adverse sensory traits while introducing more options for consumers with different needs.

A potential route to eliminating the negative sensory traits is through the addition of flavors and sweeteners (Yao et al. 2022). At the same time, the development of flavored milk can provide more options for consumers to serve different purposes. However, owing to the vast variety of PBM each with their unique taste, there is very little effort done to add flavors to the PBM. A survey by Kasapidou et al. (2023) only examined the effect of adding cocoa flavor on the nutritional, physicochemical, rheological, and antioxidant content of commercial products. Another study by Zommara et al. (2023) managed to develop chufa milk with different flavors, but the panels' size is very small (20 people) and discussions are more focused on the nutritional and physicochemical properties of the samples. A recent study by Chansuvarn and Panich (2024) found a slight increase in overall acceptance with the introduction of flavors into Sacha inchi seed milk but the increment is minute. Hence, there is a significant gap in between the development of flavored PBM and consumers' acceptance of them.

Other than flavor addition, the production and processing also have a significant impact on the sensory attributes of PBM. A study by Jaeger et al. (2024) showed two PBMs with similar raw materials and amounts of added sugar can have distinct differences in the consumers' acceptability. This further confirmed the role of processing in influencing the sensory profile of PBM. Common processing that can influence product sensory traits includes soaking, blanching, roasting, and fermentation (Ahmadian‐Kouchaksaraei et al. 2014; Tangyu et al. 2019). Subjecting raw material to roasting is a common step applied during PBM production. Roasting nuts helps to remove the raw nutty flavors while inducing a sweet, roasted aroma into the PBM through the formation of Maillard products (Zhang et al. 2016). At the same time, roasting of raw materials was found to help in reducing bitterness, beany, and chalky texture (Ahmadian‐Kouchaksaraei et al. 2014). Henceforth, there is no doubt that modification of the production process can significantly alter the sensory profile of the final product. A proper balance between processing and raw materials selection is essential to ensure a palatable PBM that supplies consumers with the required nutrients.

The raw materials used for PBM production can have a huge impact on the sensory profile of the final product. The previous experience of trying PBM from a certain raw material can significantly influence the consumers' acceptability towards another PBM from the same raw material (Yang et al. 2016). The current study utilized the seed of oil palm, palm kernel for PBM production, as it contains a complete set of essential amino acids (Kok et al. 2011) and was traditionally consumed as one of the protein sources in Nigerian's diet (Okonkwo and Ozoude 2015). However, there is no commercial use of protein in palm kernel for human consumption but only utilized as animal feed after pressing oil from palm kernel. As the second largest producer of oil palm in the world, Malaysia has a consistent supply of large quantities of palm kernel. Nevertheless, the demand for palm kernels is not as consistent as palm oil due to intense competition from coconut oil. Hence, creating a new economic use of palm kernel by converting it into a fast‐moving food product can be greatly beneficial as it creates new business opportunities for industry while enhancing food diversity and security. Other than palm kernel, pea, and rice bran protein are incorporated into the formulation as both of them have a complementary amino acid profile to palm kernel and display the advantages of hypoallergenic, cheap, and widely available (Leterme, Monmart, and Baudart 1990; Prakash 1996). The characteristic “nutty” flavor of palm kernel after blending with the “beany” pea protein and “grassy” rice bran protein creates a unique sensory profile that provides a novel taste experience for the consumers.

Therefore, the main objective of this study is to study the sensory traits in PBM that are preferred by consumers through the development of a novel palm kernel milk and to assess the effect of flavor addition in changing the consumers' preferences towards PBM. These findings are critical for providing a solid background in advancing the current trend of dairy‐free and plant‐based diets for the mission of achieving environmental sustainability and food security.

## 2 Methods and Materials

The palm kernel was sponsored by Sime Darby Plantation (Carey Island, Selangor, Malaysia). Pea protein and rice bran protein hydrolysate were purchased from MyProtein (Malaysia) and Undersun Biomedtech (Shaanxi, China), respectively. The emulsifier was provided by Futura (Pulau Indah, Selangor, Malaysia), while other food ingredients and commercial plant‐based milk (PBM) were sourced from a local bakery supply store (Bake with Yen, Selangor, Malaysia). The commercial PBMs was selected for their popularity and acceptance among local consumers, which indicates the success of their formulation in delivering desirable sensory traits. Thus, they were chosen as the commercial control in this study.

### 2.1 Production and Reformulation of Palm Kernel Milk

The formulation of palm kernel milk (PKM 1.0) was prepared according to the method described in utility innovation application number: UI 2023001869. This formulation involved incorporating vegetable oil (3.5%) and a blend of palm kernel, pea, and rice bran protein (1.6%), which provided optimal emulsion stability based on our previous study. The reformulated palm kernel milk (PKM 2.0) was prepared similarly to PKM 1.0, with slight modifications in the processing steps to reduce the raw kernel flavor and introduce a sweet aroma in the palm kernel protein extract. Improvements in PKM 2.0 include: (1) the addition of sucrose as a sweetener; (2) an increase in oil content from 3.5% to 5.0%, and (3) the incorporation of an emulsifier. For the flavored PKM 2.0 samples, flavor extracts (strawberry & vanilla) and food coloring were used to introduce the respective flavors and colors except for the chocolate‐flavored sample, where cocoa mass was used.

### 2.2 Nutritional Profile and Physical Properties

Proximate analysis was performed for all five samples and commercial PBM (unflavored oat milk, almond milk, soymilk). Protein analysis was performed using the Kjeldahl method with a nitrogen conversion factor of 5.83, 5.18, 5.50, and 6.25 (Chee, Ling, and Ayob 2012; Food and Agriculture Organization, 2003; House et al. 2019) for oat milk, almond milk, palm kernel milk (PKM), and soy milk, respectively. Lipid content was analyzed using the modified Bligh and Dyer method with methanol and chloroform replaced with isopropanol and hexane (Moneeb et al. 2021). Moisture and ash content were determined according to AOAC methods 972.20 and 945.46, respectively. Carbohydrate content was calculated gravimetrically using the formula:

$$\begin{array}{cc} \text{Carbohydrate content}\left(\%\right)\; & =100\%-\text{protein content}\\ & -\text{lipid content}-\text{moisture content}\\ & -\text{ash content} \end{array}$$

Lightness, L*, redness, a* and yellowness, b* of all samples were measured using a color spectrophotometer (Colorflex EZ, Hunterlab, Melbourne, Australia). The viscosity was measured using an MCR 302 modular compact rheometer (Anton Paar, Graz, Austria) with a concentric ring (20 mm diameter) at 25°C by varying shear rate from 0.01 s^−1^ to 100 s^−1^. The flow curves obtained were then analyzed using the Newtonian model. The total soluble solid was determined using a refractometer (Master Series, Atago, Japan).

### 2.3 Sensory Profiling

Two rounds of sensory evaluation studies were conducted for PKM 1.0 and PKM 2.0 samples. Panelists were recruited randomly utilizing campus‐wide posters, flyers, and classroom announcements. During the evaluations, panelists were provided with water to cleanse their palates between samples and asked to rate their preferences based on attributes such as appearance, aroma, taste, mouthfeel, and overall preference. A nine‐point hedonic scale was used, along with a check‐all‐that‐apply (CATA) analysis based on 20 descriptive keywords (see Table 1). Panelists were instructed to select all keywords relevant to the sensory attributes of the samples provided. Although the panelists were not trained in professional sensory evaluation, they were briefed on the process and definitions of the keywords to ensure comprehension.

**TABLE 1:** Total response for each keyword in the CATA analysis by the 100 panelists in first and second round of sensory evaluation.

	Soymilk	Oat milk	Almond milk	PKM 1.0	PKM 2.0	Vanilla	Strawberry	Chocolate
Appearance								
Whitish	28	7	95	5	66	55	2	0
Yellowish	76	80	2	8	16	19	0	1
Dull	10	30	11	78	48	47	7	16
Greasy	7	15	8	19	22	14	6	17
Aroma								
Beany	63	43	48	38	50	41	17	11
Earthy	16	24	22	48	34	31	9	13
Grassy	10	5	17	14	9	6	10	1
Nutty	40	60	41	35	45	54	12	50
Roasted	15	35	5	31	24	28	2	35
Taste								
Astringent	6	10	8	38	15	14	17	5
Bland	8	9	87	27	41	19	21	3
Rich	72	77	1	26	25	34	10	79
Sweet	67	46	3	3	35	61	59	64
Artificial	9	11	11	28	31	26	66	10
Mouthfeel								
Chalky	9	14	18	39	28	18	20	18
Creamy	75	80	4	25	46	34	19	75
Coarse	1	4	1	11	7	5	8	13
Oily	9	8	4	22	20	15	8	8
Smooth	68	66	50	32	45	57	46	60
Watery	8	13	82	36	32	57	65	8

#### 2.3.1 Round 1

A total of 100 panelists, comprising 68% females and 32% males aged 18–47 were recruited for the study. PKM 1.0 was compared against three different commercial plant‐based milk samples; almond milk, oat milk, and soy milk. In addition to the nine‐point hedonic and CATA tests, panelists were asked to indicate their frequency of plant‐based milk consumption and to suggest potential flavors (e.g., chocolate, fruits, matcha) for further sensorial enhancements in PKM 1.0.

#### 2.3.2 Round 2

A sensory acceptance test was conducted on the improved formulation developed based on the responses from the Round 1 sensory evaluation. A new group of 100 panelists, comprising 70% females and 30% males aged 18–47, was recruited for this round. PKM 2.0 was served alongside three other PKM samples with added flavoring agents representing the top three preferred flavors from the first round: vanilla, strawberry, and chocolate. Panelists were provided with a similar hedonic and CATA questionnaire.

### 2.4 Statistical Analysis

All analyses were performed in triplicate and presented as mean ± standard deviation (SD). A one‐way ANOVA followed by Tukey's post hoc test (*p* < 0.05) was conducted to analyze the proximate and physicochemical properties of the samples using GraphPad Prism version 10.0.2 for Windows (GraphPad Software Inc., Boston, Massachusetts, USA). To ensure data normality assumptions were met, a D'Agostino‐Pearson test was performed prior to the ANOVA analysis.

CATA results were analyzed using R studio (RStudio, PBC, Boston) (R Core Team 2023). A principal component analysis (PCA) was conducted on the CATA keyword frequency table and the corresponding hedonic score using the “prcomp” function. A biplot was generated with the factoextra package (version 1.0.7) (Kassambara and Mundt 2020). Additionally, a correlation matrix combining both CATA and hedonic results was created using the corrplot package (version 0.92) (Wei and Simko 2021) with a 5% significance level.

## 3 Result and Discussion

### 3.1 Sample Characterization

Among the commercial samples, soymilk contained the highest protein, followed by oat milk and almond milk (Table 2). This trend aligns with findings reported by Aydar, Tutuncu, and Ozcelik (2020). Interestingly, oat milk was found to have a fat content comparable to soymilk, whereas almond milk was very low in fats. The relatively high fat content in oat milk in this study is likely due to the addition of fat in the commercial product formulation, as oats naturally contain much less fat than soybeans or almonds. This observation is supported by Pointke et al. (2022), who reported that average oat products on the market had lower fat content than almond milk and soy milk. Compared to commercial plant‐based milks (PBMs), PKM 1.0 showed the highest fat content and the lowest carbohydrate content, with its protein content second only to that of soymilk. The lower carbohydrate content in PKM 1.0 compared to other commercial PBMs, is due to the absence of sweeteners or gums, which are commonly added to other PBMs to enhance flavor and texture.

**TABLE 2:** Proximate and physical properties of soymilk, oat milk, almond milk, and PKM samples.

	Commercial PBM			Palm kernel milk				
	Soymilk	Oat milk	Almond milk	PKM 1.0	PKM 2.0			
					Original	Vanilla	Strawberry	Chocolate
Protein (%)	2.45 ± 0.02^a^	0.73 ± 0.01^b^	0.67 ± 0.11^b^	0.98 ± 0.05^c^	0.86 ± 0.02^b,c^	0.93 ± 0.02^c^	1.00 ± 0.01^c^	1.65 ± 0.01^d^
Fat (%)	2.23 ± 0.02^a^	2.29 ± 0.22^a^	1.20 ± 0.02^b^	3.58 ± 0.07^c^	4.61 ± 0.36^d^	3.84 ± 0.20^c,e^	4.11 ± 0.07^e^	5.17 ± 0.13^f^
Moisture (%)	88.67 ± 0.03^a,d^	87.63 ± 0.05^a^	97.18 ± 0.01^b^	94.74 ± 0.16^c^	88.98 ± 0.03^d,f^	90.69 ± 0.02^e^	89.97 ± 0.03^e,f^	83.46 ± 0.02^g^
Ash (%)	0.53 ± 0.10^a^	0.84 ± 0.02^b^	0.01 ± 0.01^c^	0.12 ± 0.02^d^	0.06 ± 0.01^c,d^	0.02 ± 0.00^c^	0.05 ± 0.01^c,d^	0.60 ± 0.01^a^
Carbohydrates (%)	6.12 ± 0.13^a,d^	8.51 ± 0.21^b^	0.94 ± 0.14^c^	0.58 ± 0.15^c^	5.49 ± 0.36^d^	4.52 ± 0.20^e^	4.87 ± 0.08^d,e^	9.12 ± 0.13^b^
Viscosity (mPa.s)	5.20 ± 0.05^a^	15.21 ± 0.79^b^	1.60 ± 0.01^c^	1.47 ± 0.07^c^	1.88 ± 0.05^c^	1.50 ± 0.00^c^	1.54 ± 0.01^b^	16.25 ± 0.24^d^
Color								
L*	83.08 ± 0.01^a^	80.28 ± 0.04^b^	89.52 ± 0.02^c^	78.25 ± 0.11^d^	85.27 ± 0.01^e^	83.73 ± 0.16^f^	78.23 ± 0.43^d^	39.95 ± 0.08^g^
a*	2.46 ± 0.01^a^	3.49 ± 0.01^b^	0.75 ± 0.01^c^	5.30 ± 0.06^d^	2.49 ± 0.01^a^	2.91 ± 0.04^a^	22.05 ± 0.81^e^	13.56 ± 0.04^f^
b*	13.46 ± 0.01^a^	14.1 ± 0.01^b^	5.84 ± 0.01^c^	10.67 ± 0.08^d^	10.12 ± 0.01^e^	11.71 ± 0.36^f^	8.30 ± 0.39^g^	15.10 ± 0.06^h^
Total soluble solid (°Brix)	11.5 ± 0.0^a^	13.5 ± 0.0^b^	2.5 ± 0.0^c^	5.5 ± 0.0^d^	8.5 ± 0.0^e^	10.0 ± 0.0^f^	10.5 ± 0.0^g^	14.0 ± 0.0^h^

*Note:* Different superscripts within the same row differ significantly (*p* < 0.05).

With the reformulation of PKM 2.0, there was a significant increase (*p* < 0.05) in fat and carbohydrate content due to the addition of vegetable oil and sugar. The addition of flavorings (strawberry and vanilla) did not significantly alter the nutritional content of PKM, except in the chocolate‐flavored version. In this case, cocoa mass was used instead of a cocoa flavor extract, contributing additional protein, carbohydrates, and fats. These results align with findings by Kasapidou et al. (2023), who reported significant nutritional changes in PBMs with the addition of cocoa.

The analysis of color (L*, a*, b*) serves as an indirect, objective measurement for vision aspects in sensory evaluation. Among the commercial samples, almond milk exhibited the highest lightness (L*), consistent with findings by Jemma et al. (2023). The high whiteness in almond milk may result from its low soluble solid content, which minimally affects the color of the emulsion. In contrast, soymilk and oat milk, with higher soluble solid content, displayed a darker, more yellow, and red hue compared to almond milk. PKM 1.0 had the lowest whiteness, likely due to uneven distribution of oil droplets (data not shown); varying droplet sizes can reduce light scattering. Additionally, natural dark brown color of palm kernel protein contributed to a yellower and redder sample. With reformulation, PKM 2.0 showed a significant increase in whiteness (L*) and a decrease in redness (a*), attributed to added vegetable oil and an emulsifier, which helped disperse oil droplets. In flavored samples, the addition of food flavoring and coloring altered the color profile, shifting it away from a whitish tone.

The rheological behavior of PBMs is closed related to their composition. Oat milk and chocolate‐flavored PKM, which have the highest carbohydrate content, exhibited significantly higher viscosity. This trend is attributed to the presence of starch and soluble fiber in oat and cocoa mass, which gelatinize or act as viscosifying agent upon heat treatment, thereby increasing PBM viscosity (Deswal, Deora, and Mishra 2014). This explains the minimal increase in viscosity observed in other PKM 2.0 samples, where the carbohydrate increase is due to added of sugar rather than starch. Additionally, the inclusion of food flavoring and coloring was found to marginally reduce the viscosity of PKM 2.0.

### 3.2 Hedonic Score

**FIGURE 1:**
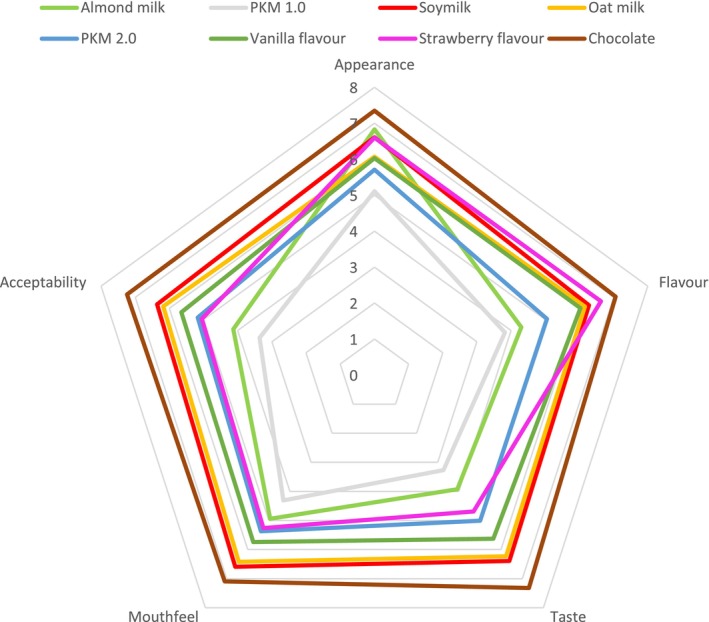
Hedonic score for appearance, aroma, taste, mouthfeel, and acceptability of the eight samples in first and second round of sensory evaluation.

The sensory evaluation results showed that PKM 1.0 was the least liked sample across all attributes compared to other commercial PBMs in the first round, with an average acceptability score of 3.36 out of 9 (Figure 1). Soymilk was the most preferred sample (6.36 out of 9), followed by oat milk (6.18 out of 9), while almond milk ranked second least preferred (4.13 out of 9). Only two products received a positive response, suggesting that most PBMs had a low consumer preference. This result aligns with Yao et al. (2022), who found that most PBM products received acceptability scores between 5 and 6 out of 9, and Jaeger et al. (2024), who observed that 9 out of 18 PBMs scored below 5 out of 9. However, Jemma et al. (2023) reported at opposite trend, with almond milk as the most preferred PBM, likely due differences in PBM brand used in their study. The higher preference for commercial samples highlights the maturity of their formulations and suggests potential directions for improving PKM 1.0.

A reformulation of PKM was conducted based on the preferred attributes of soy and oat milk (to be discussed in Section 3.3.1). Compared to PKM 1.0, the reformulated PKM 2.0 achieved a higher acceptance score of 5.17 out of 9, indicating a shift in consumer preferences and the effectiveness of reformulation. The addition of sugar and flavoring improved PKM acceptability across samples, except for the strawberry‐flavored PKM. Rincon, Botelho, and de Alencar (2020) reported similar improvements in aroma and taste when vanilla extract was added to coconut and chickpea milk, supporting Yao et al. (2022)'s prediction that introducing flavors and sweeteners can enhance PBM acceptability. The lower acceptance of the strawberry‐flavored PKM may be due to a mismatch between its vivid red appearance, intense aroma, and a slight chemical aftertaste. Overall, the chocolate‐flavored PKM received the highest acceptability score of 7.24 out of 9. Consumer preference for cocoa‐flavored products over other flavors is consistent in other studies (Chansuvarn and Panich 2024; Zommara et al. 2023).

### 3.3 CATA Analysis

All samples showed a mixed response, suggesting that none were perceived as highly similar (Table 1). Briefly, the CATA responses for “whitish”, “yellowish”, and “sweet” aligned well with results from the colorimeter and total soluble solid content (Table 2). In terms of mouthfeel, there was less agreement in describing low‐viscosity samples with terms like “creamy” and “watery”; however, samples with significantly higher viscosity were generally rated higher for “creamy” and lower for “watery”. This is consistent with findings by Wang et al. (2021), who noted that creamier samples tend to exhibit higher viscosity, though the difference may not always be significant. Upadhyay and Chen (2019) proposed that perceived mouthfeel and viscosity may differ due to variations in emulsion systems, which can impact both bulk and localized rheology. A low response frequency was found for the appearance attributes of strawberry‐ and chocolate‐flavored PKM, indicating that the keywords set may not adequately describe these samples, as added food dyes drastically changed their appearance from typical PBM.

#### 3.3.1 PCA Analysis

**FIGURE 2:**
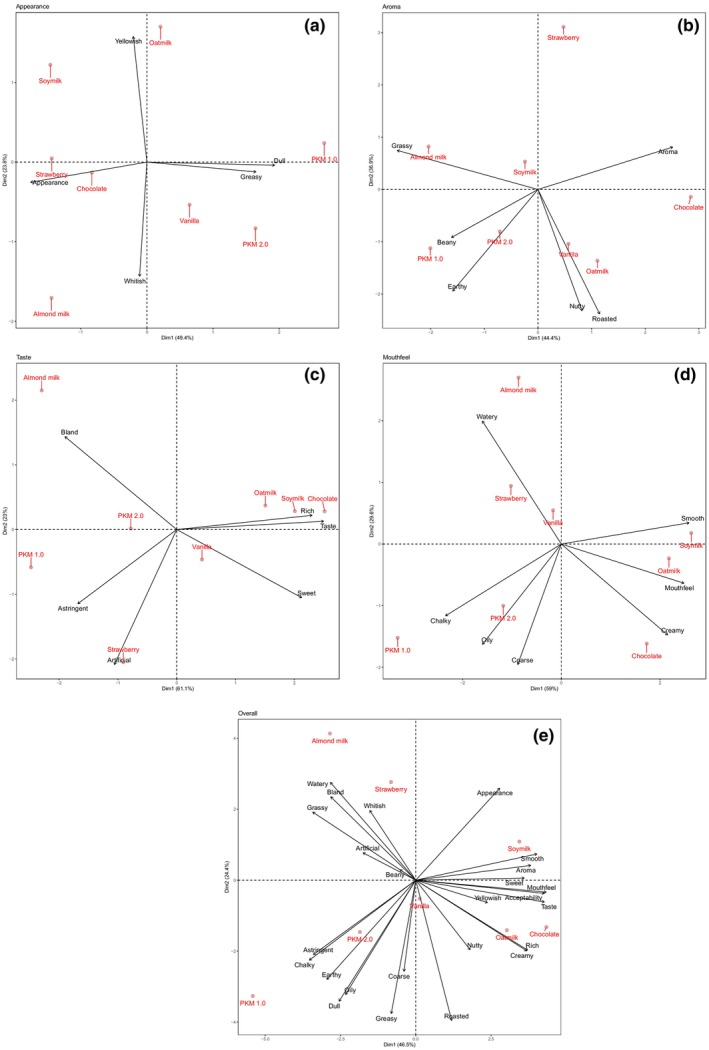
Sensory attributes: appearance (a), aroma (b), taste (c), mouthfeel (d), overall (e) representation of samples based on first two principal components.

Data from the CATA evaluation was analyzed using principal component analysis (PCA) to visualize how PBM samples are described with CATA keywords. The PCA included individual attributes (appearance, aroma, taste, and mouthfeel) along with the overall acceptance score. Hedonic analysis results were also incorporated to explore potential correlations between specific sensory attributes and their hedonic preferences. In all PCA analysis, the combined contribution of PC1 and PC2 accounted for over 70% of the variance, indicating a good model fit. PCA results (Figure 2e) revealed that none of the samples were closely grouped, confirming that each sample was distinct.

##### 3.3.1.1 Appearance

The appearance of a product is the first attribute that attracts consumers to try a new item. For milk products, a lighter color is generally more appealing, though excessive lightness beyond typical dairy can be off‐putting (Oduro, Saalia, and Adjei 2021). PKM 1.0 was not described as “whitish” or “yellowish” (Figure 2a), which aligns with its low L* and high a* values. Instead, PKM 1.0 was strongly described as “dull” and “greasy”, unlike the commercial samples, which suggests their formulation are optimized for a more attractive appearance. After reformulation, PKM 2.0 shifted significantly to the left and bottom of the graph, indicating a less pronounced dull and greasy appearance and a lighter, whiter color. This change was due to the addition of emulsifiers and fats, which allowed the oil to disperse more evenly, creating a homogenous, whiter emulsion. Similarly, a study on lentil milk found an increase in whiteness with higher oil content (Jeske et al. 2019). The addition of food dye and cocoa mass improved the appearance significantly, as shown by the position of the strawberry‐ and chocolate‐flavored samples on the far left of PC1. This shift suggests that the current CATA keywords may be inadequate to describe these samples' appearance, consistent with previous observations. The spread of “yellowish” and “whitish” along PC2 suggests a low correlation with the appearance's hedonic score, likely because the inclusion of flavored samples reduced the relative importance of “whitish” in determining preference.

##### 3.3.1.2 Aroma

Aroma refers to the sensory perception consumers receive through sniffing a product. For PKM 1.0, the aroma profile (Figure 2b) is mainly described as “beany”, “earthy”, and “grassy”. This aligns with palm kernel's volatile compounds, which contribute green, fruity, herbal and earthy notes (Zhang et al. 2016), and the addition of pea and rice bran proteins further enhances the “beany” and “grassy” aromas. The “beany” aroma, defined as a raw soybean scent, is often considered an undesirable off‐flavor in PBMs (Day N'Kouka et al. 2004). Among commercial samples, both almond and soymilk displayed stronger “grassy” aromas, followed by “beany” notes. This association is likely due to the presence of aldehyde, compounds known to impart “grassy” scents (Pointke et al. 2022). However, this differs from other findings, such as Moss et al. (2022), who identified almond milk primarily “white”, “fruity”, and “no aftertaste” characteristics. Soymilk is typically noted for its strong “beany” aroma, as seen in other studies (Jaeger et al. 2024; Yao et al. 2022), with “grassy” aromas secondary. This discrepancy is likely due to the flavoring agents added on the commercial formulation that may alter the aroma profile.

Oat milk showed a similar aroma profile to vanilla‐flavored PKM, characterized by “nutty” and “roasted” notes. The “roasted” aroma likely results from the increase in sulfur compounds during cereal processing, producing a toasted effect (Vaikma et al. 2021).

Reformulation shifted PKM 2.0 significantly towards a more neutral aroma, likely due to an increased vegetable oil content that helps mask intense flavors leading to a blander profile. Additionally, the modified processing in PKM 2.0 contributed a sweet and nutty aroma to the palm kernel, enhancing the final product's aroma. Conversely, the strawberry‐ and chocolate‐flavored PKM samples were not characterized by typical PBM attributes, indicating that added flavors completely altered their aroma profiles.

##### 3.3.1.3 Taste

Taste is a key sensory attribute influencing consumers preference for certain products. A study by Collier et al. (2023) found that the perceived superior taste of dairy milk is a primary reason consumers reject plant‐based milk. PKM 1.0 was strongly associated with a astringent taste (Figure 2c), which likely contributed to its low hedonic score, as shown by the contrasting positions of “astringent” and “taste” on PC1. This finding aligns with Day N'Kouka et al. (2004) and Lesme et al. (2024), who concluded that astringency is a major factor driving negative consumer perceptions. In this study, almond milk displayed a distinct taste profile, described as “bland”, while oat and soymilk shared similar profiles, described as “rich” and “sweet”. None of the commercial samples showed a correlation with “astringent”, suggesting that the absence of this characteristic contributes to their market acceptance. Since astringency is a strong negative trait in PBM, a reformulation was undertaken to reduce this attribute in PKM 2.0. This involved process modification to remove off‐flavors and the addition of sugar and flavoring to introduce a sweeter taste.

A 50% reduction in the “astringent” response for PKM 2.0, compared to PKM 1.0 (Table 1), was observed alongside a significant rightward shift along PC1 in Figure 2c. This aligns with findings by Geburt et al. (2022), who concluded that adding sugar can mask bitter flavors in plant‐based milk, thereby increasing acceptability relative to unsweetened versions. Similarly, vanilla flavor and cocoa mass enhanced PKM 2.0's acceptability. Vaikma et al. (2021) reported that vanillin effectively masks strong astringent aftertaste, while McClure, Hopfer, and Grün (2022) noted that consumers generally tolerate slight bitterness in chocolate products (McClure, Hopfer, and Grün 2022). For the strawberry‐flavored PKM, the high concentration of flavor extract seemed to mask the astringency, though it introduced an “artificial” taste that contributed to a lower hedonic score. This suggests that optimizing flavor extract concentration is crucial to prevent off‐flavors. Overall, the process modifications successfully reduced off‐flavors in PKM while the addition of sugar and flavors effectively masked remaining astringency, enhancing consumer acceptability.

##### 3.3.1.4 Mouthfeel

Mouthfeel, or texture, refers to the physical sensation of food in the mouth. For oil‐in‐water emulsions, it often associated with the friction coefficient between food components, saliva, and the salivary pellicle coating the oral surface (Lesme et al. 2024). The mouthfeel of plant‐based milk generally aligns with descriptors like “watery” and “chalky” (Collier et al. 2023) or “lumpy” and “thick” (Vaikma et al. 2021), influenced by the raw materials and formulation. In this study, PKM 1.0 was predominantly characterized as “chalky”, “oily”, and “coarse” (Figure 2d), which consumers found undesirable. None of the commercial samples occupied the same sensory quadrant as PKM 1.0. Almond milk was more closely associated with a “watery” mouthfeel, while oat and soymilk were generally described as “creamy” and “smooth”.

Studies by Wang et al. (2021) suggest that creaminess negatively correlates with particle size, while Chen (2015) found that perceived creaminess increases with droplet concentration. Additionally, smoothness is closely related to the lubricating effect of components in the emulsion, which is significantly enhanced by a higher oil mass fraction (Upadhyay and Chen 2019). Therefore, the reduction of “chalky” and “coarse” sensation in PKM 2.0 can be attributed to its higher oil content which provides greater lubrication. Furthermore, the introduction of an emulsifier facilitates the formation of smaller oil droplets, further enhancing the creaminess of PKM 2.0.

For the vanilla‐ and strawberry‐flavored PKM, the dominant characteristic was “watery” indicates a change in mouthfeel perception upon addition of flavoring. This alteration may be attributed to the emulsifier present in the flavoring, which affects the physicochemical behavior of the sample on the oral surface. Interestingly, the chocolate‐flavored PKM is positioned close to oat and soymilk, characterized by a “creamy” and “smooth” mouthfeel. This enhancement in creaminess and smoothness can likely be traced to the cocoa butter residue in the cocoa mass introduced through the addition of cocoa powder.

### 3.4 Correlation Matrix Analysis

#### 3.4.1 CATA Keywords

**FIGURE 3:**
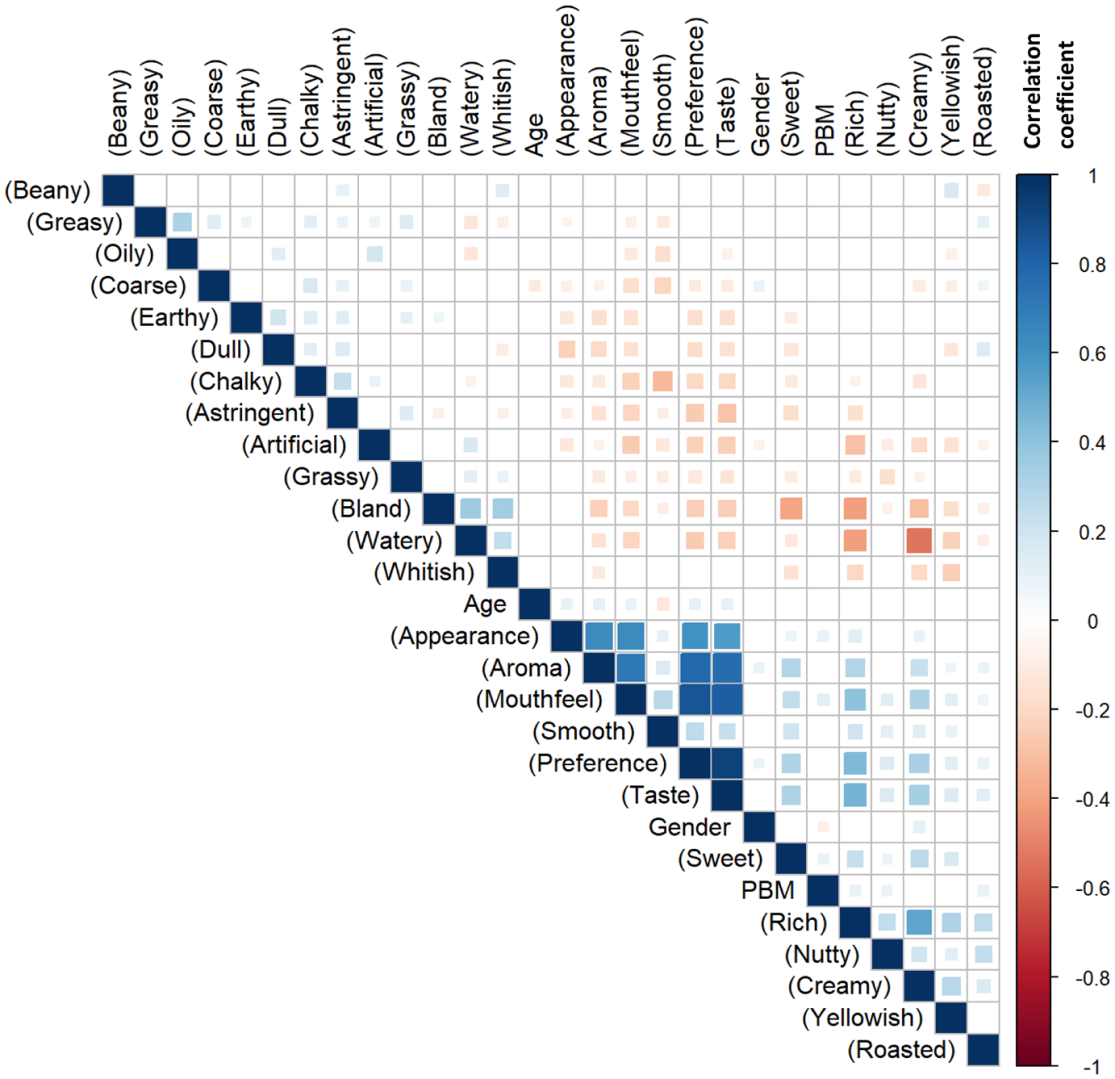
Correlation matrix of CATA keywords, panelists' background, and sensory attributes hedonic score. Only statistically significant correlations (*p* < 0.05) were included. For panelists' background, a value was assigned to each response with a higher value assigned to a higher age group or frequency of PBM consumption. For gender, female was assigned as 1 and male was assigned as 2.

Despite the potential to infer the correlation between CATA attributes with the hedonic acceptance score from the PCA biplot, the results are not conclusive and may lack statistically significant (*p* < 0.05). To gain a clearer understanding of the potential correlations between the CATA keywords and their corresponding sensory attributes, a correlation plot was generated, focusing on those correlations that were statistically significant (*p* < 0.05) (Figure 3).

A “dull” appearance (*R* = −0.8930) and a “greasy” appearance (*R* = −0.5329) strongly and moderately contribute to lower appearance scores, respectively. This finding aligns with expectations, as “dull” and “greasy” attributes lead to a less appealing product. However, in terms of color, the classification of products as either “whitish” or “yellowish” does not significantly influence consumer preferences for product appearance. This finding contrasts slightly with results from Cardello et al. (2022), Jaeger et al. (2024), and Oduro, Saalia, and Adjei (2021), which report a weak but significant correlation between “whitish” sample and consumer acceptance. The discrepancy may be attributed to the inclusion of flavored sample in this study, which appears to diminish the impact of “whitish” coloration on consumer acceptance.

Conversely, “earthy” (*R* = −0.7782) and “grassy” (*R* = −0.8026) aromas exhibit a negative, moderate to strong correlation with the hedonic score of sample aroma, while “roasted” (*R* = 0.0809) shows a very weakly correlation. Additionally, both “nutty” and “beany” sensations do not appear to significantly influence the hedonic score for product aroma. This finding contrasts with Yang et al. (2016), who reported that soymilk with a less intense beany flavor received higher acceptability ratings. The authors noted that preferences might be significantly affected by the cultural background of the panelists. Similarly, Jaeger et al. (2024) found both positive and negative perceptions of the “nutty” flavor in PBM, likely due to consumers segmentation. While roasting is known to reduce the “beany” flavor in soymilk (Day N'Kouka et al. 2004), the low correlation observed here may be attributed to the absence of a strong roasted aroma in any of the samples.

The presence of “astringent” (*R* = −0.7347), “bland” (*R* = −0.6689) and “artificial” (*R* = −0.4080) attributes in the product's taste profile moderately decreases taste preferences. In contrast, “rich” (*R* = 0.8534) and “sweet” (*R* = 0.8285) flavors strongly enhance taste preferences. As mentioned, “astringent” is a significant negative taste attribute that adversely affects consumers' acceptability of plant‐based products (Lesme et al. 2024), aligning with its highest negative correlation coefficient. Sweetness, on the other hand, is an innate human preference (Demattè, Endrizzi, and Gasperi 2014). The term “artificial” typically describes unexpected or undesirable intense flavors. A study by Collier et al. (2023) indicated that consumers who do not regularly consume PBM often associate “artificial” flavor with negative connotations. The terms “rich” and “bland” represent opposite ends of flavor spectrum, and it is not surprising that a bland flavor diminishes consumers preference, especially in beverages expected to offer distinct taste and flavor. Supporting this, Cardello et al. (2022) found that a “weak/bland” flavor negatively impact the average liking of a product.

Regarding mouthfeel, attributes such as “coarse” (*R* = −0.0208), “oily” (*R* = −0.4664), “watery” (*R* = −0.6920) and “chalky” (*R* = −0.7443) negatively impact consumer preferences, with correlations strength ranging from weak to moderate. Conversely, “creamy” (*R* = 0.8635) and “smooth” (*R* = 0.8872) are highly preferred mouthfeel characteristics, exhibiting strong positive correlations. The description of mouthfeel effectively reflects the quality of PBM emulsions; “oily”, “watery”, “chalky”, and “coarse” sensations often indicate poorly formulated emulsions, potentially leading to oil–water separation and large particulates sedimentation. In contrast, “creamy” and “smooth” mouthfeel suggest a homogeneous and well‐formulated emulsion. This aligns with findings by Cardello et al. (2022), where ‘creamy mouthfeel’ and ‘thick/viscous’ sensations are among the most positively received sensory attributes. Palacios et al. (2009) described this preference as characteristic of a ‘cream liker’, who favors strong milk aromas alongside creamy and smooth textures. However, Jaeger et al. (2024) reported no correlation between “thick/viscous” and consumer liking, suggesting that a balanced mouthfeel may be more crucial than merely achieving creaminess.

An analysis of the correlation between various sensory attributes and consumer acceptance on the hedonic scale reveals a decreasing trend: taste (*R* = 0.9961) = mouthfeel (*R* = 0.9854) > aroma (*R* = 0.8874) > appearance (*R* = 0.6128). This indicates that taste and mouthfeel are more influential in determining consumer acceptance than aroma and appearance. This finding contrasts with the conclusions of Yao et al. (2022), who noted that consumers' overall liking primarily stemmed from aroma and taste, rather than texture and appearance. Nevertheless, Andersen, Brockhoff, and Hyldig (2019) indicated that consumers often struggle to discriminate between retro‐nasal (oral) and ortho‐nasal (sniff) aromas, which may diminish the perceived importance of aroma in influencing overall acceptability.

Sensory attributes that negatively affect consumers' overall acceptability include “coarse”, “earthy”, “dull”, “chalky”, “astringent”, “artificial”, “grassy”, “bland”, and “watery”. In contrast, attributes that enhance consumers' acceptability encompass: “smooth”, “sweet”, “rich”, “nutty”, “creamy”, “yellowish”, and “roasted”.

#### 3.4.2 Panelists' Background

In addition to product characteristics, the potential influence of panelists' backgrounds on sensory acceptance was also analyzed as summarized in Table 3. It is important to note that the sample sizes of the different background groups vary significantly, which may explain why all significant correlations found exhibit weak strength. There is a significant but weak, positive correlation between age and the hedonic score of all products served (Figure 3), indicating that older panelists tend to have higher acceptability for various products.

**TABLE 3:** Panelists' background involved in first and second round of sensory analysis.

	1st round of sensory study	2nd round of sensory study
Age group		
18–23	64	76
24–29	21	17
30–35	12	3
36–41	2	3
42–47	1	1
Gender		
Male	32	30
Female	68	70
Frequency plant‐based milk consumption		
Less than once per month	58	57
Once per month	17	19
Once per week	8	12
More than once per week	17	12

In terms of gender, male participants generally gave higher score for aroma and product preference than females participants, although the correlation remains weak. The correlations between age and gender with acceptability towards different PBMs can be explained by the degree of food neophobia. Individuals with higher food neophobia tends to have a narrower range of acceptable food consumed and vice versa (Demattè et al. 2013; Demattè, Endrizzi, and Gasperi 2014). In general, older individuals and women are expected to be more receptive to novel tastes and flavors (Endrizzi et al. 2019). Nevertheless, the impact of individual background on food neophobia, particularly concerning age and gender, remains debated due to discrepancies in findings across studies (Demattè et al. 2013; Demattè, Endrizzi, and Gasperi 2014).

Given the uneven sample sizes in this study, the results suggest potential attitudes of different demographics towards a new flavor of plant‐based milk. Interestingly, there is no correlation between the frequency of PBM consumption and preference for the samples, indicating that prior experience with PBM does not significantly influence an individual's sensory acceptance of a new product. This is different from the result of Jaeger et al. (2024), who found a positive correlation between the frequency of PBM consumption and acceptance score. The discrepancy may arise from the use of novel palm kernel milk in this study, which minimizes the possibility of previous exposure affecting acceptability. Additionally, the small sample size of individuals who regularly consumed plant‐based milk may also impact the result.

### 3.5 Limitation and Suggestions for Future Research

This study has identified several limitations. First, the overrepresentation of younger panelist (ages 18–23) may have skewed the sensory preference results, particularly in favor of a higher acceptance of “sweet” taste, as this age group often exhibits a stronger preference for sweetness (Yeo et al. 2022). Future studies with a more diverse age range would better reflect the general population and provide a more accurate assessment of product acceptance.

Additionally, this study did not investigate how food neophobia might influence consumer acceptance of the PKM sample. Although a weak correlation between age, gender, and sample preference was discovered, this relationship could be influenced by varying levels of food neophobia within the population. Since exploring food neophobia was beyond the scope of this study, future research should examine its impact on the acceptance of novel plant‐based milks to validate this potential correlation.

## 4 Conclusion

In conclusion, this study successfully identified the sensory traits of plant‐based milk (PBM) that consumers prefer through the development and reformulation of a novel palm kernel milk (PKM) using hedonic and CATA sensory analysis. The sensory attributes found to contribute positively to consumer preference include “smooth”, “sweet”, “rich”, “creamy”, and “roasted.” Process reformulation effectively reduced the undesirable “beany” aroma, while improvements in formulation—such as the addition of emulsifiers, sugar, and oil—resulted in a whiter, creamier emulsion with a sweet taste that consumers favor.

Furthermore, the introduction of various flavors into PKM significantly altered its organoleptic profile, effectively masking negative flavors such as “beany,”, “grassy”, and “astringent.” Among the samples, chocolate‐flavored PKM emerged as the most acceptable due to its rich, sweet taste, coupled with a creamy and smooth mouthfeel. This finding highlights the potential of flavor addition in enhancing consumer acceptability.

The results of this study offer a new direction for modifying the sensory profiles of PBM to improve consumer acceptance. Notably, the lack of correlation between previous consumption experience and consumer acceptance suggests that PBMs with entirely novel tastes are judged fairly by consumers, independent of their prior experiences with other PBMs. Overall, this study lays the groundwork for developing a novel, nutritious PBM that is well‐accepted by consumers, contributing to a more diverse and sustainable vegetarian market.

## Conflicts of Interest

The authors declare no conflicts of interest.

## Data Availability

The data that support the findings of this study are available on request from the corresponding author. The data are not publicly available due to privacy or ethical restrictions.
